# Destructive Effect of Intravitreal Heat Shock Protein 27 Application on Retinal Ganglion Cells and Neurofilament

**DOI:** 10.3390/ijms21020549

**Published:** 2020-01-15

**Authors:** Pia Grotegut, Sandra Kuehn, H. Burkhard Dick, Stephanie C. Joachim

**Affiliations:** Experimental Eye Research Institute, University Eye Hospital, Ruhr-University Bochum, In der Schornau 23-25, 44892 Bochum, Germany; Pia.Grotegut@ruhr-uni-bochum.de (P.G.); Sandra.Kuehn@ruhr-uni-bochum.de (S.K.); Burkhard.Dick@kk-bochum.de (H.B.D.)

**Keywords:** glaucoma, heat shock protein 27 (HSP27), retinal ganglion cell degeneration, cell stress

## Abstract

Heat shock protein 27 (HSP27) is commonly involved in cellular stress. Increased levels of HSP27 as well as autoantibodies against this protein were previously detected in glaucoma patients. Moreover, systemic immunization with HSP27 induced glaucoma-like damage in rodents. Now, for the first time, the direct effects of an intravitreal HSP27 application were investigated. For this reason, HSP27 or phosphate buffered saline (PBS, controls) was applied intravitreally in rats (*n* = 12/group). The intraocular pressure (IOP) as well as the electroretinogram recordings were comparable in HSP27 and control eyes 21 days after the injection. However, significantly fewer retinal ganglion cells (RGCs) and amacrine cells were observed in the HSP27 group via immunohistochemistry and western blot analysis. The number of bipolar cells, on the other hand, was similar in both groups. Interestingly, a stronger neurofilament degeneration was observed in HSP27 optic nerves, while no differences were noted regarding the myelination state. In summary, intravitreal HSP27 injection led to an IOP-independent glaucoma-like damage. A degeneration of RGCs as well as their axons and amacrine cells was noted. This suggests that high levels of extracellular HSP27 could have a direct damaging effect on RGCs.

## 1. Introduction

Glaucoma, besides age-related macular degeneration and diabetic retinopathy, ranges among the most common causes for irreversible vision loss worldwide. These numbers will further increase by 2030 [1]. Glaucoma is considered a multifactorial disease, which is defined by a progressive loss of retinal ganglion cells (RGC) and degeneration of the optic nerve [2]. The causative pathomechanisms are still ill-defined. However, current research projects focus on different mechanisms, which are suspected to contribute to glaucomatous damage. These include high intraocular pressure [3], oxidative stress [4], and excitotoxicity [5] but also immunological processes. Several studies demonstrated that glaucoma patients have altered autoantibody titers against different proteins and antigens including phosphatidylserine [6], neuron-specific enolase [7], S100B [8], and heat shock proteins (HSPs) [9,10,11].

HSPs are ubiquitously expressed, have a highly conserved structure, and are induced in response to various physiologic and environmental stressors. They perform chaperone functions by stabilizing new proteins and helping to refold proteins that were damaged by different types of stress [12]. HSP27 is also known as heat shock protein beta-1 (HspB1) or HSP25. It is one of the smallest HSPs and is most commonly involved in stress [13]. For example, after optic nerve transection (mechanical stress and neuronal stress) as well as whole-body hyperthermia, HSP27 expression was increased in the rat hippocampus and the visual system [14,15]. Chidlow et al. investigated the expression level of HSP27 in four different retinal degeneration models. They used a model of acute axonal optic nerve crush, an *N*-methyl-d-aspartate (NMDA)-induced excitotoxicity model, an ocular hypertension glaucoma model (OHT), and a model of chronic hypoperfusion through bilateral occlusion of the carotids. In all models, an increased expression of HSP27 at the protein and mRNA level could be detected in the retina [16]. In the case of glaucoma, there is evidence that HSP27 may play an important role in the pathomechanisms. Glaucoma patients demonstrated elevated serum titers of antibodies against HSP27 [9,11]. Additionally, an increased HSP27 level could be observed in retinas of glaucoma patients [17]. For this reason, the role of HSP27 in various models was investigated [18,19]. On one hand, HSP27 has a protective effect and prevented apoptosis in some experimental models. It was described that intracellular HSP27 binds cytochrome c and prevents cytochrome c mediated interaction of Apaf-1 with procaspase 9 in human leukemic cell line U937 [20]. Furthermore, HSP27 is a stabilizer of the cytoskeleton by protecting actin filaments during oxidative stress. This experiment was performed in Chinese hamster CCL39 cells [21]. Yokoyama et al. described protective effects on RGCs after electroporation of HSP27 into the vitreous in an ischemia-reperfusion model [22]. On the other hand, HSP27 suppression initiated a protective effect on photoreceptor cells in a light-induced retinal degeneration model [23]. In previous studies by Wax et al. and our group, systemic immunization of HSP27 resulted in glaucomatous damage [24,25]. In addition, the effect of HSP27 depends on its localization. Extracellular HSP27 is presumed to have other functions than intracellular HSP27. Studies suggests that extracellular HSP27 serves as a signaling molecule released by immune cells, while intracellular HSP27 is viewed as a protective and anti-apoptotic protein [26]. HSP27 is most likely released by the endolysosomal pathway and through exosomes [26]. Depending on the model and localization of HSP27, the protein may have both protective and degenerative effects.

In this study, we investigate the direct effects of an intravitreal HSP27 injection on the retina and the optic nerve. Here, we noted a degeneration of RGCs, amacrine cells, and the optic nerve neurofilament after HSP27 injection. We assume that the high, non-phosphorylated extracellular level of HSP27 activated cell death mechanisms.

## 2. Results

### 2.1. Stable Intraocular Pressure and only Slight Effects in Retinal Signal Transmission

**Figure 1:**
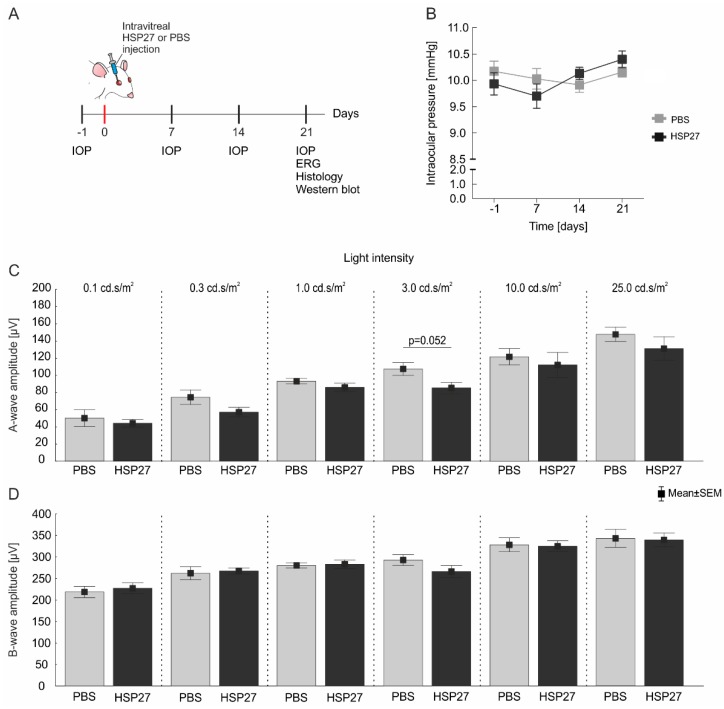
HSP27 immunization had no effect on retinal function or intraocular pressure. (**A**) The IOP was measured before (−1 day) as well as 7, 14, and 21 days after the intravitreal injection. HSP27 or its solvent phosphate buffered saline (PBS) were intravitreally injected at day 0. The electroretinogram (ERG) examination as well as the histological and western blot analyses were performed after 21 days. (**B**) No differences in IOP between HSP27-injected animals and controls could be found before the intravitreal injection (−1) or after 7, 14, and 21 days (*p* > 0.09). (**C**) No significant changes in the ERG a-wave amplitude were detected in HSP27 and PBS eyes (*p* > 0.05). Only a trend to a decrease was noted in HSP27 eyes at 3.0 cd.s/m^2^ (*p* = 0.052). (**D**) Likewise, no differences in the b-wave amplitude were found between both groups (*p* > 0.1). *n* = 6/group.

Intraocular pressure (IOP) was measured weekly before and for three weeks after the injection (Figure 1A). No differences between HSP27-injected eyes and control eyes were present at all points in time (all: *p* > 0.09, Figure 1B).

The retinal functionality was investigated via electroretinogram (ERG) recordings after 21 days. No significant changes were found in the amplitude of a-wave (Figure 1C) and b-wave (Figure 1D) of the ERG measurements in the HSP27 animals compared to the PBS animals. Only a trend was found at 3.0 cd.s/m^2^ (HSP27: 85.1 ± 6.6 μV, PBS: 107.3 ± 7.613 μV, *p* = 0.052) in the a-wave amplitude (Figure 1C).

### 2.2. Intact Retinal Morphology but Observable Cell Loss

**Figure 2:**
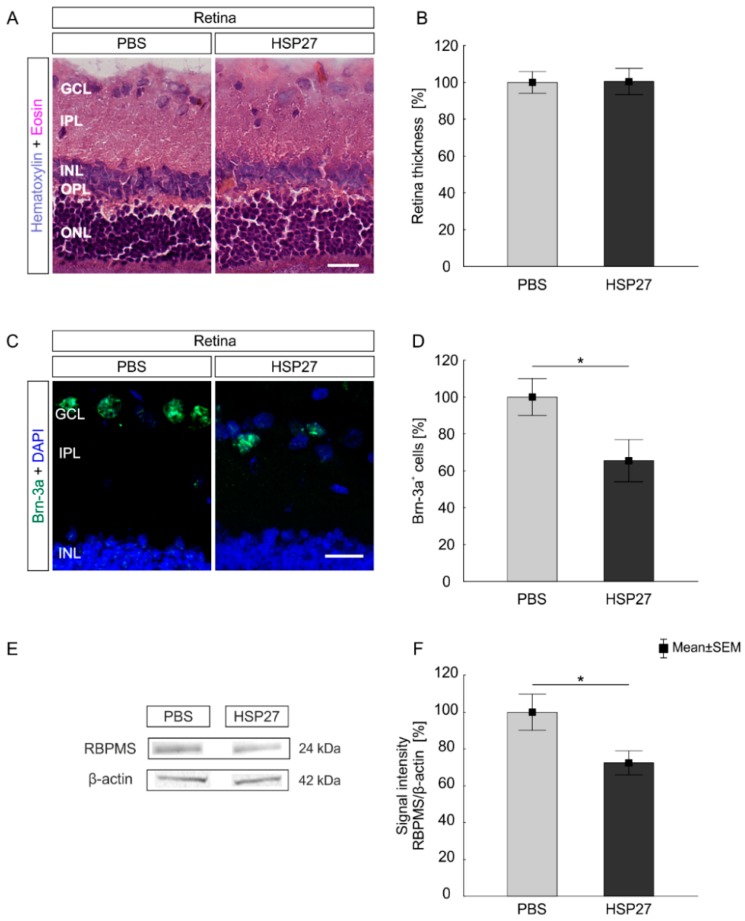
No changes in retinal morphology, but observable neurodegeneration. (**A**) Retinas from HSP27 and PBS animals were stained with HE. (**B**) After 21 days, no differences in the retina thickness were noted between the HSP27 and PBS group (*p* > 0.05). (**C**) Retinal ganglion cells (RGCs) in the retina were marked with Brn-3a (green) and cell nuclei with 4′,6-Diamidin-2-phenylindol (DAPI, blue). (**D**) Brn-3a cell count revealed an RGC loss in the HSP27 group (*p* = 0.046). (**E**) The protein level of RNA-binding protein with multiple splicing (RBPMS; 24 kDa) was measured with western blot and normalized with β-actin (42 kDa). (**F**) The western blot analysis of RBPMS demonstrated a significant lower protein amount in the HSP27 group (*p* = 0.04). GCL = ganglion cell layer, IPL = inner plexiform layer, INL = inner nuclear layer, OPL = outer plexiform layer, ONL = outer nuclear layer, scale bar = 20 µm, *n* = 6/group (immunohistology), *n* = 5/group (western blot), * *p* < 0.05.

Haematoxylin and eosin (HE)stained retinas were evaluated (Figure 2A). The integrity of the retina remained intact after intraocular injections of HSP27 (100.5 ± 7.1%) as no differences in the thickness of the retina was found compared to the PBS group (100.0 ± 5.9%; *p* > 0.9; Figure 2B).

To analyze neuronal degeneration after HSP27 injection in more detail, we quantified the number of RGCs using Brn-3a, a specific RGC marker (Figure 2C). The cell counts of Brn-3a^+^ cells demonstrated a decrease in the in the RGC number in HSP27 group (65.5 ± 11.4%) compared to the PBS group (100.0 ± 9.9%, *p* = 0.04; Figure 2D). To verify RGC degeneration, western blot analyses were performed using the specific RGC marker RNA-binding protein with multiple splicing (RBPMS; Figure 2E) [27]. The RBPMS protein level was significantly lower in the HSP27 group (72.4 ± 6.5%) than in the PBS group (100 ± 9.75%, *p* = 0.04; Figure 2F).

### 2.3. Degeneration of the Inner Retina Structures

**Figure 3:**
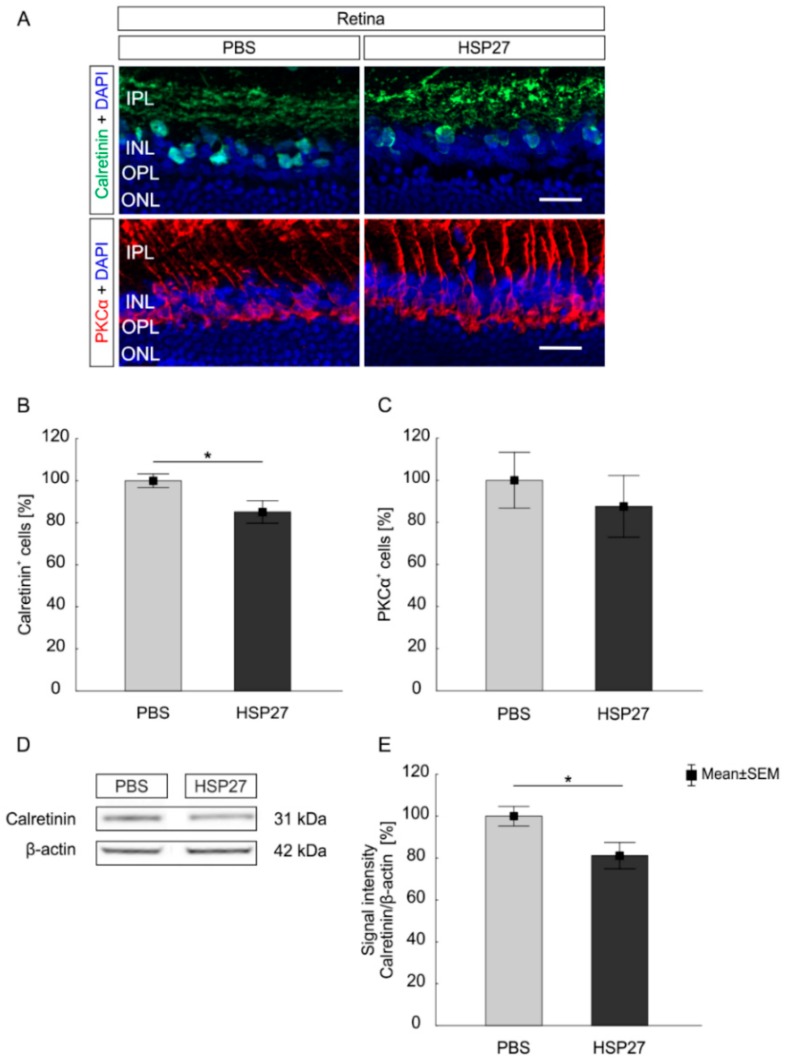
Degeneration in the inner retina layer. (**A**) The amacrine and bipolar cells were stained with the antibody calretinin (green) and PKCα (red). 4’,6-Diamidin-2-phenylindol (DAPI; blue) was used to label cell nuclei. (**B**) Fewer calretinin^+^ amacrine cells were noted in the HSP27 group (*p* = 0.038). (**C**) The number of bipolar cells was similar in both groups (*p* > 0.05). (**D**) Calretinin (55 kDa) was analyzed via western blot using β-actin (42 kDa) for normalization at day 21. (**E**) A lower calretinin level was detected in the HSP27 group compared to the PBS group (*p* = 0.031). IPL = inner plexiform layer, INL = inner nuclear layer, OPL = outer plexiform layer, ONL = outer nuclear layer, scale bar = 20 µm, *n* = 6/group (immunohistology and western blot), * *p* < 0.05.

In addition to RGCs, amacrine cells and bipolar cells were analyzed to investigate the impact of HSP27 on neuronal cells of the retina. The number of amacrine cells, stained with an anti-calretinin antibody, was significantly lower in the HSP27 group (85.1 ± 5.3%) compared to the PBS group (100.0 ± 3.2%, *p* = 0.03; Figure 3A,B). Western blot analysis confirmed the loss of calretinin in HSP27 injected retinas (81.1 ± 6.2%) compared to the PBS group (100.0 ± 4.6%, *p* = 0.03; Figure 3D,E).

In contrast, bipolar cells, visualized with PKCα, were not affected by the HSP27 injection (Figure 3A). Similar numbers of PKCα^+^ cells were counted in the HSP27 group (87.6 ± 14.7%) and the control group (100.0 ± 13.3%, *p* = 0.5; Figure 3C).

#### HSP27 Injection Did Not Lead to Retinal Glia Cell Activation

**Figure 4:**
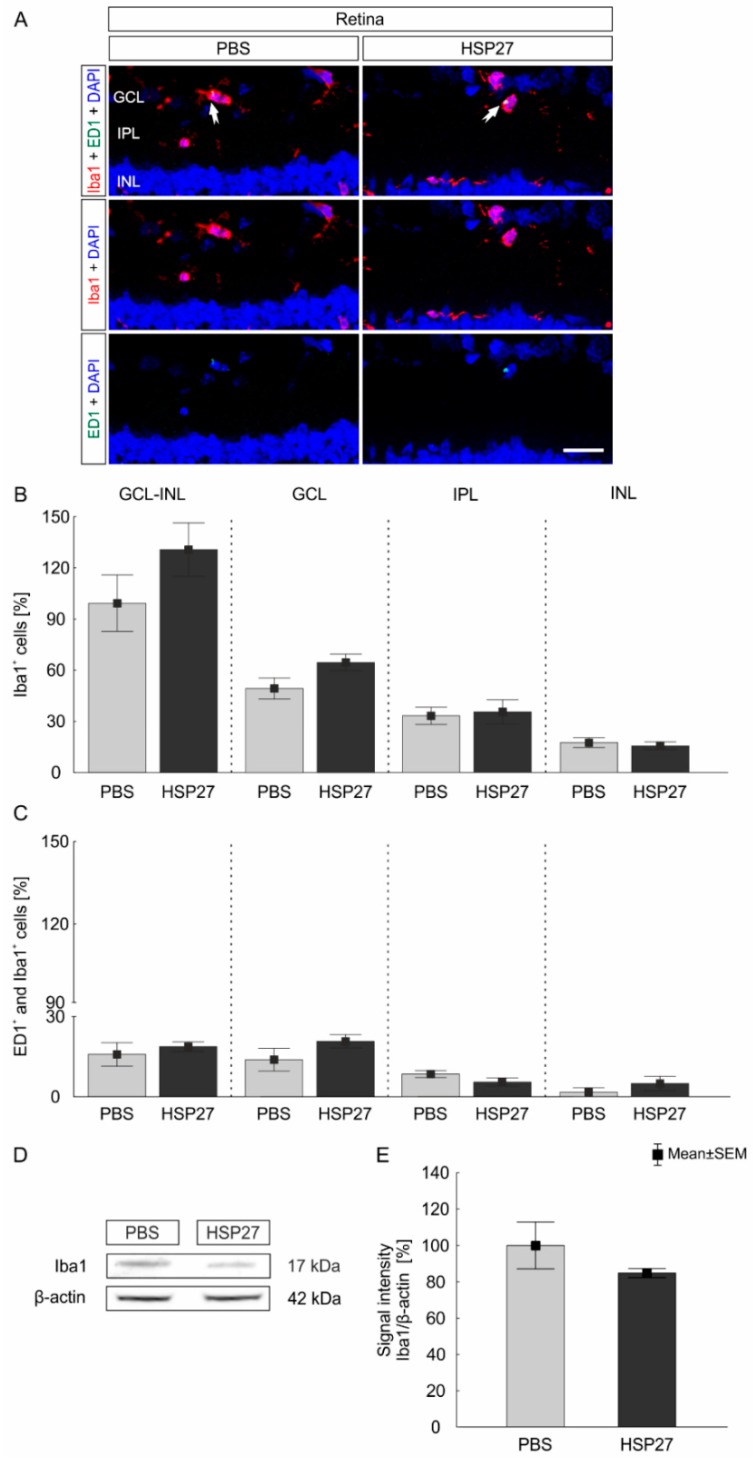
No microglia/macrophages cell response in retina after HSP27 injection. (**A**) Iba1 (red) and ED1 (green) were used to identify microglia/macrophages and active ones (arrows) in the retina. The cell nuclei were stained with 4’,6-Diamidin-2-phenylindol (DAPI; blue). (**B**) The number of Iba1^+^ cells was similar in the HSP27 group and the PBS group in the GCL, IPL, and INL (*p* > 0.05). In addition, the total cell count of Iba1^+^ cells for the layers from GCL to INL together showed no differences between the groups (*p* > 0.2). (**C**) Furthermore, the number of ED1^+^ and Iba1^+^ cells, hence active microglia/macrophages, was almost the same in both groups in each analyzed layer and in the area from the GCL to the INL (*p* > 0.07). (**D**) The protein level of Iba1 (17 kDa) was determined by western blot and normalized with β-actin (42 kDa). (**E**) The Iba1 protein level remained unchanged at day 21 in comparison to the control group (*p* = 0.3) GCL = ganglion cell layer, IPL = inner plexiform layer, INL = inner nuclear layer, scale bar = 20 µm, *n* = 6/group (immunohistology and western blot).

The effect of intravitreal HSP27 injection on glial cells, in particular macroglia and microglia, was also analyzed in the retina after 21 days. Iba1 is a protein that is specifically expressed in the cells of monocytic lineages, including microglia and macrophages [28]. The antibody against transmembrane protein 119 (Tmem 119) was additionally used to detect just microglia cells, since it is a specific marker for microglia and nor for macrophages [29]. The whole microglia/macrophage population (Iba1^+^) and the amount of activated microglia/macrophages (ED1^+^ and Iba1^+^) were investigated (Figure 4A). The number of Iba1 as well as ED1^+^ and Iba1^+^ cells were counted from the GCL to the INL. Regarding the number of Iba1^+^ microglia/macrophages in GCL up to INL, no differences could be observed in the HSP27 group (130.6 ± 15.7%) compared to controls (100.0 ± 16.6%; *p* = 0.2; Figure 4B) In the PBS group, 49.2 ± 15.04% of Iba1^+^ cells were present in the GCL, 33. 2 ± 12.3% in the IPL and 17. 5 ± 7.1% in the INL. In the HSP27 group, 64.4 ± 11.8% (*p* = 0.07) Iba1^+^ signals were counted in the GCL, 35.4 ± 17.3% (*p* = 0.79) in the IPL, and 15.6 ± 2.4% (*p* = 0.53) in the INL. No significant differences between the groups could be detected in these layers (Figure 4B).

Furthermore, similar numbers of activated (ED1^+^ and Iba1^+^) microglia/macrophages were observed in the PBS (20.1 ± 2.9%) and the HSP27 group (24.2 ± 1.8%; *p* = 0.22; Figure 4C). Similar results could be demonstrated for the individual retinal layers. The count of ED1^+^ and Iba1^+^ signals showed 13.8 ± 10.4% in the GCL of PBS control retinas, 8.4 ± 3.1% in the IPL and 1.6 ± 3.7% in the INL. Through the HSP27 injection similar results were presented. The largest proportion of ED1^+^ and Iba1^+^ signals was detected in the GCL (20.7 ± 6.2%, *p* = 0.19), while lower cell counts were detected in the IPL (5.4 ± 2.1%, *p* = 0.07) and INL (4.8 ± 6.6%, *p* = 0.36). Again, no significant difference between the two groups could be detected in these cell layers (Figure 4C). Moreover, possible alterations of Iba1 protein level were analyzed via western blot (Figure 4D). In accordance with the immunohistochemical results, the amount of Iba1 protein was comparable in the HSP27 (84.9 ± 1.3%) and the PBS group (100.0 ± 12.8%; *p* = 0.3; Figure 4E).

**Figure 5:**
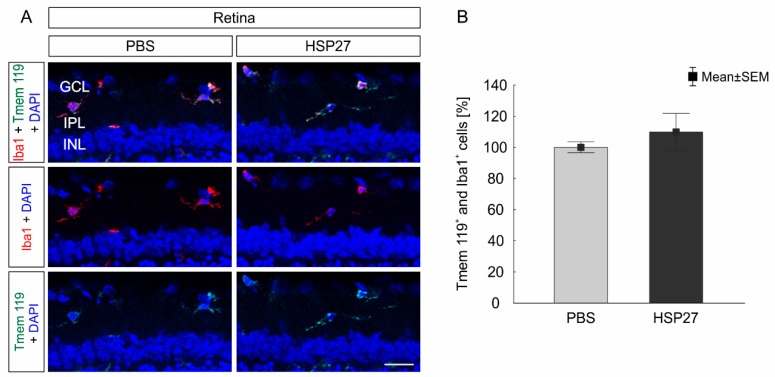
No microglia response in retina after HSP27 injection. (**A**) Antibodies against Iba1 (red) and transmembrane protein 119 (Tmem 119, green) were used to identify the retinal microglia population. The cell nuclei were stained with 4’,6-Diamidin-2-phenylindol (DAPI; blue). (**B**) The numbers of Tmem 119^+^ and Iba1^+^ cells were similar in the HSP27 group and the PBS group (*p* = 0.4) GCL = ganglion cell layer, IPL = inner plexiform layer, INL = inner nuclear layer, scale bar = 20 µm, *n* = 6/group.

Microglia cells were stained with the markers Tmem 119 and Iba1 (Figure 5A). No differences were detected between the PBS (100.0 ± 3.5%) and the HSP27 group (109.5 ± 12.1%, *p* = 0.4; Figure 5B).

**Figure 6:**
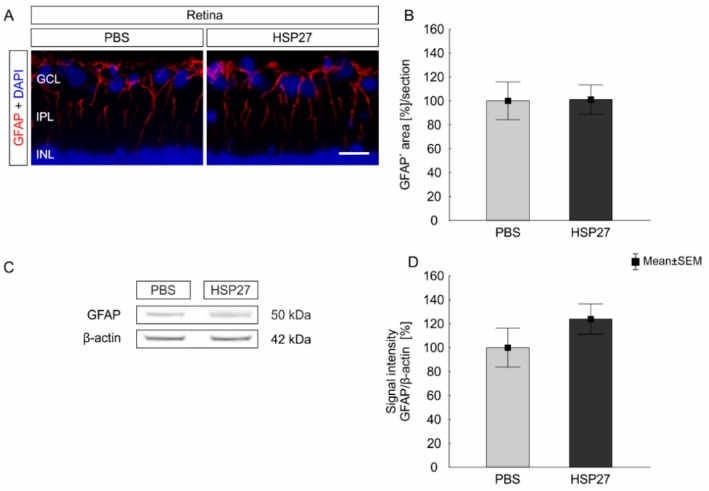
No macroglia cell response after HSP27 injection. (**A**) Macroglia (GFAP, red) and cell nuclei (DAPI, blue) were stained in the retina. (**B**) After 21 days, the GFAP^+^ area of the HSP27 group was similar to the one in the PBS group (*p* = 0.83). (**C**) GFAP (50 kDa) protein level was measured with western blot and normalized with β-actin (42 kDa). (**D**) Again, no significant differences were found between the two groups (*p* = 0.27). GCL = ganglion cell layer, IPL = inner plexiform layer, INL = inner nuclear layer, scale bar = 20 µm, *n* = 6/group (immunohistology and western blot).

To visualize macroglia, an antibody against GFAP was used (Figure 6A). However, the GFAP^+^ area in the HSP27 (101.1 ± 12.3%) and the PBS group (100.0 ± 15.8%, *p* = 0.83; Figure 6B) was quite similar. In accordance with these findings, the western blot analysis revealed no differences in the GFAP protein amount after HSP27 (123.9 ± 12.6%) and PBS injection (100.0 ± 16.2%; *p* = 0.27; Figure 6C,D).

### 2.4. No Demyelination, but Neurofilament Damage of Optic Nerves, after 21 Days

**Figure 7:**
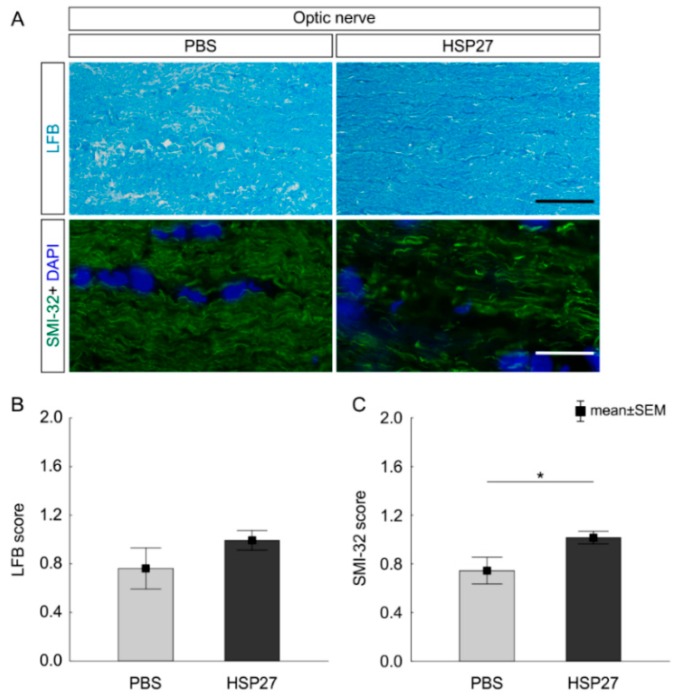
Intact myelin sheath and damaged neurofilament. (**A**) LFB was used to stain the optic nerve myelin sheaths. The antibody against SMI-32 (green) was applied to identify the neurofilaments in the optic nerve. The cell nuclei were stained with DAPI (blue). (**B**) 21 days after the HSP27 injection, no effects on the myelin were noted via LFB scoring (*p* = 0.27). (**C**) The SMI-32 score revealed a degeneration of neurofilaments in the HSP27 group (*p* = 0.049). Scale bar = 20 µm, *n* = 6/group, * *p* < 0.05.

Myelin sheaths of optic nerves were stained with luxol fast blue (LFB) 21 days after the intravitreal injection (Figure 7A). No differences between the PBS group (0.7 ± 0.2 mean score) and HSP27 group (0.9 ± 0.1 mean score, *p* = 0.21) could be detected regarding the LFB score (Figure 7B). Hence, HSP27 likely had no damaging effect on the myelin sheaths of the optic nerves.

However, neurofilament damage was detected. Nerves were stained with anti-SMI-32 antibody to visualize possible disruptions of the axonal neurofilaments (Figure 7A). In contrast to the PBS group (0.7 ± 0.1 mean score), optic nerves of the HSP27 group (1.0 ± 0.1 mean score) showed an increased SMI-32 score and therefore a degeneration of neurofilaments (*p* = 0.049; Figure 7C).

### 2.5. No Glia Cell Response in the Optic Nerve

**Figure 8:**
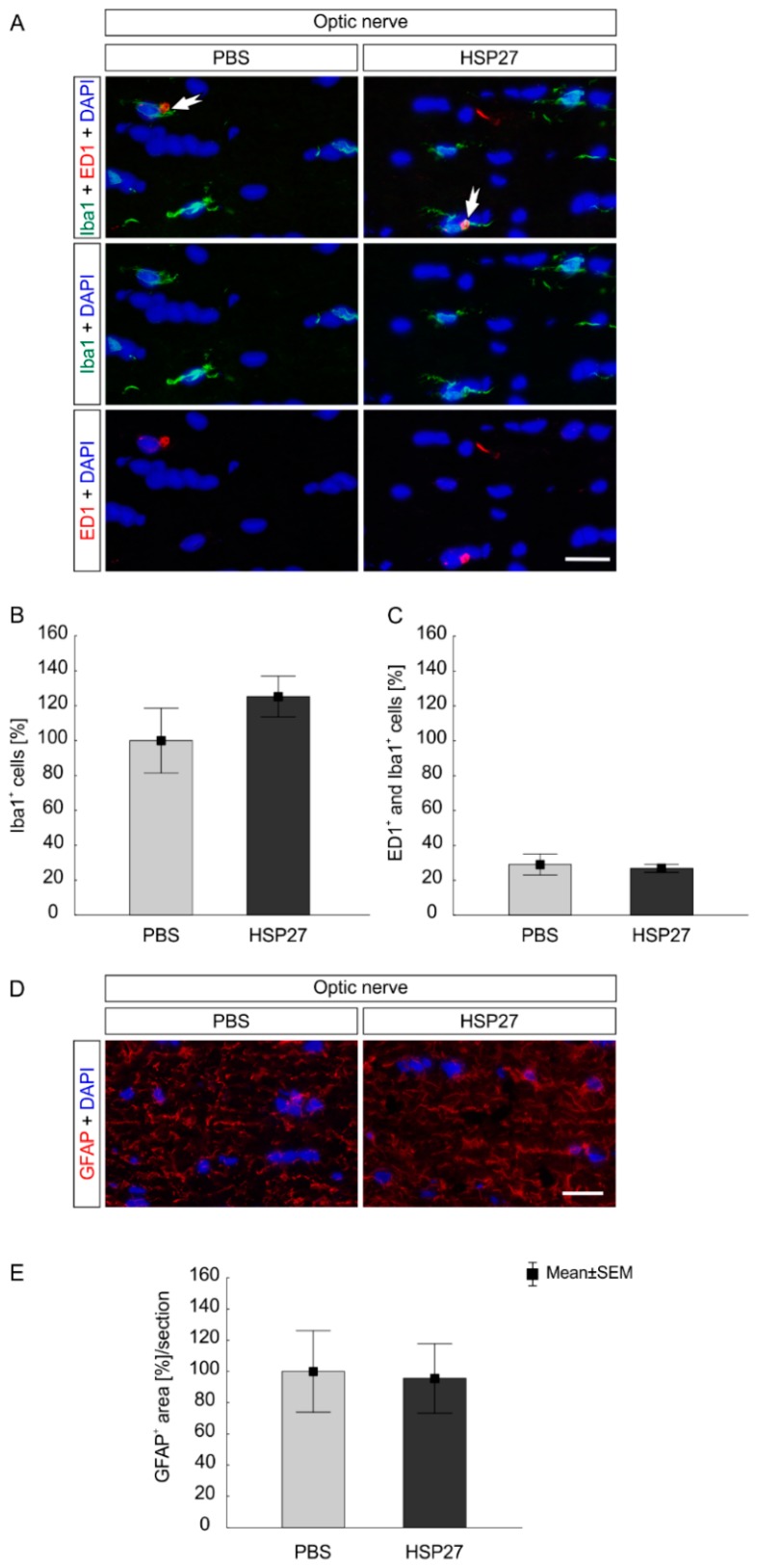
Optic nerve demonstrated no glia cell response after HSP27 injection. (**A**) Iba1 (green), ED1 (red), and DAPI (blue) were stained in optic nerves to evaluate microglia/macrophages and activated ones (arrows). (**B**) The number of Iba1^+^ microglia/macrophages was similar in the HSP27 and the PBS group (*p* = 0.27). (**C**) The same was noted for the number of activated microglia /macrophages (ED1^+^ and Iba1^+^). No differences could be observed between both groups (*p* = 0.73). (**D**) Macroglia (GFAP, red) and cell nuclei (DAPI, blue) were stained in the optic nerve. (**E**) Additionally, there was no difference in GFAP^+^ area between the HSP27 group and the PBS group (*p* = 0.9). Scale bar = 20 µm, *n* = 6/group.

Microglia/macrophages activity was also investigated in the optic nerves (Figure 8A). Concerning the total population of microglia/macrophages, cell counts revealed similar numbers of Iba1^+^ microglia/macrophages in optic nerves of the HSP27 group (125.2 ± 11.7%) and the PBS control group (100.0 ± 18.6%; *p* = 0.27; Figure 8B). In consistence with the findings in the retina, the number of activated microglia/macrophages (ED1^+^ and Iba1^+^ cells) did not differ between the investigated groups (PBS 29.0 ± 5.9%; HSP27 26.8 ± 2.2%, *p* = 0.73; Figure 8C).

The macroglia response was analyzed using GFAP staining in optic nerve tissue (Figure 6D). No differences between the PBS (100.0 ± 26.0%) and HSP27 group (95.6 ± 22.2%) were found (*p* = 0.9; Figure 8E). Hence, optic nerve glial cells were not activated after HSP27 injection.

## 3. Discussion

HSP27 was intravitreally injected to investigate possible effects on cells in retina and optic nerve. In the recent years, studies described an increased concentration of HSP27 in the retina of glaucoma patients [17]. Furthermore, in different glaucoma animal models, high levels of HSP27 were observed in the retina [18,19]. Therefore, HSP27 likely plays a role in glaucoma pathomechanisms, but this conclusion needs to be further investigated. In this study, we could identify a degeneration of RGCs and amacrine cells in the retina. Also, degeneration of the optic nerve neurofilament was observed after intravitreal HSP27 injection. These results provide evidence for degenerative effects of extracellular HSP27.

### 3.1. Stable Intraocular Pressure

Several studies described that an intraocular injection of small volumes does not cause an increased intraocular pressure [30,31]. This was also the case after the intravitreal injection of HSP27. The IOP stayed in normal ranges throughout the study. Also, the protein itself did not induce an IOP elevation.

### 3.2. Ganglion Cell Loss Did Not Affect the Total Retinal Structure

At day 21, the thickness of the retina was unaltered and its general structure remained intact, while we found a significant loss of RGCs. These results are in accordance with previous studies, where a systemic immunization with HSP27 induced RGC loss after five weeks [24,25]. RGCs degenerated earlier after a direct application in the eye. A fast degeneration was also detectable in models based on intravitreally injected S100B, TNF-α, or *N*-Methyl-d-aspartic acid (NMDA) [32,33,34]. In contrast to this degenerative effect, several studies demonstrated that HSP27 prevented the degeneration of neuronal cells through stabilization of the actin cytoskeleton or the suppression of apoptotic stimuli [22,35]. In addition, the downregulation of HSP27 led to apoptosis of human retinal endothelial cells [36]. Hence, HSP27 can demonstrate degenerative and protective effects in different models or conditions. It might be possible that the role of HSP27 is dose dependent. S100B, a glial cell stress protein, on the one hand, induced neuroprotective properties in low concentrations, but on the other hand, it caused neurodegeneration in high concentrations [37,38,39]. In this context, the function of HSP27, as a stress protein, could be similar. Another cause for the degenerative effect of HSP27 could be that it is an effective anti-inflammatory regulator in a phosphorylated state [40]. Also, the necessary MAP kinases act intracellularly, whereas the intravitreal injection rather enhanced the extracellular amount of HSP27. Non-phosphorylated HSP27 increased the ubiquitin-mediated degradation of cAMP-response element-binding protein (CREB-binding protein), which is an important protein to reduce inflammatory processes. In addition, extracellular HSP27 serves as a signaling molecule. Some membrane receptors have been identified for extracellular HSP, including cluster of differentiation (CD)91, CD40, CD36, CD14, toll-like receptors (TLR), and scavenger receptor-A [41]. In mouse coronary endothelial cells, HSP27 interacts with TLR-2 and TLR-4 inducing nuclear factor κB (NF-κB) phosphorylation [42]. NF-κB can control several cellular mechanisms, including proliferation, survival, and apoptosis [43].

Another theory for the degeneration is the involvement of the immune system. Previously, in sera of glaucoma patients, an elevated antibody titer against HSP27 was detected [9]. Wax et al. described that HSP27 might serve as an antigenic stimulus by activating the innate and/or adaptive immune response during glaucomatous neurodegeneration. In this case, HSP27 acts as a danger signal and high HSP27 levels help to detect and eliminate stressed RGCs through the immune system. Hence, an uncontrolled immune response may facilitate the progression of the neurodegeneration [24]. In a recent study, the IOP elevation led to upregulation of membrane bound and extracellular HSPs in the ganglion cell layer. This upregulation induced immune mediated neural damage through activation of the HSP-specific CD4^+^ T-cell response [44]. This indicates that HSP27 could act as an antigen stimulus to initiate an immune response. This could also be the case in our study, where a high level of HSP27 was present in the eye.

### 3.3. Further Degeneration and Slightly Changed Retinal Electrophysical Response

To examine the degeneration more closely, the other neurons of inner retinal layers were also examined, more specifically amacrine and bipolar cells. Both are interneurons of the retina and important for visual signal transfer, since they modulate and transfer signals to the RGCs [45]. We noted a loss of amacrine cells in the HSP27 group. Similar observations were recently made by our group, when S100B was injected intravitreally [34]. Also, after systemic immunization of HSP27 in combination with S100B, a degeneration of amacrine cells was noted [46]. Moreover, in glaucoma animal models, like the DBA/2J mouse model or high pressure models, a reduction of amacrine cells was also described [47,48]. A study suggested that amacrine cells are volatile to glaucoma-like damage and that amacrine cell damage occurs secondarily to RGC death through gap-junction-mediated bystander effects [49]. This could also be the case in our study, since we noted the loss of RGCs and amacrine cells. However, the ERG measurements showed no significant changes in the b-wave amplitudes, which reflect the electrical signal transmission in the INL. The loss of the amacrine cells does not seem to have any influence on the functionality of the electrical signal transmission at this time. Perhaps, the amacrine cell death was still in the initial phase at that point in time and sufficient amacrine cells were present to ensure accurate signal transmission. In contrast, the bipolar cells were not affected after the intravitreal HSP27 application. This finding is in accordance with intravitreal injected S100B or NMDA studies [34,50]. Furthermore, the immunization of HSP27 together with S100B did not impair PKCα^+^ bipolar cells. Only recoverin^+^ cone bipolar cells were affected [46]. In a glaucoma model with elevated IOP, significantly fewer rod bipolar cells were noted [51]. In our model, no massive degeneration of bipolar cells was observed. It might also be possible that the degeneration process is reduced or inhibited because bipolar cells are not as sensitive to the loss of RGCs as amacrine cells. Interestingly, HSP27 animals showed a slight degeneration in the a-wave amplitude at 0.3 cd.s/m^2^, pointing towards a small damage to signal transmission of the photoreceptor cells. This was also observed for 1 cd.s/m^2^ after the intravitreal injection of S100B [34]. Chien et al. described that HSP27 is necessary to protect photoreceptors during light exposure, but that it should be suppressed after light exposure to promote cell recovery [23]. Perhaps, high levels of HSP27 might be disruptive for healthy photoreceptors. In contrast, a strong decrease of electrical signal transmission could be noted after induction of retinal ischemia, which induces an intense damage in the whole retina [52].

### 3.4. Wallerian-Like Degeneration of the Optic Nerve

In order to investigate the effect of intravitreally injected HSP27 on the optic nerve, the neurofilament and the myelin sheaths were analyzed. A degeneration of the neurofilament was noted, while the myelin sheaths remained unaffected. Since we observed retinal degeneration, axonal degeneration in the present study is possibly the result of direct injury to the cell bodies of the RGCs. This type of degeneration is also called Wallerian-like degeneration. Wallerian-like degeneration [53] was also described in different glaucoma animal models and after intravitreal application of certain substances, like kainic acid [54]. However, the important point here is that degeneration of the neurofilaments was observed. There is evidence that the RGC axons within the optic nerve head are the primary site of damage in glaucoma [2,55]. In the human optic nerve head, the lamina cribrosa consists of fibro elastic lamellae and is lined by astrocytes that support the non-myelinated RGC axons as they exit the eye. In glaucoma, the morphology of the lamina cribrosa is altered [56]. Furthermore, the expression of HSP27 was increased in reactive astrocytes in the lamina cribrosa of glaucoma patients [17]. For this reason, an elevated HSP27 level could affect the neurofilament degeneration in the optic nerve in humans. However, we do not yet know where the initial site of degeneration is. Likely, the degeneration of RGCs appears prior to the optic nerve damage. This was also described for the intravitreal injection of NMDA [50]. Currently, we cannot exclude that the degeneration starts in the optic nerve and subsequently leads to a retrograde retinal cell body damage at later points in time. This course of damage was previously described for the intravitreal injection of TNF-α and S100B [32,34]. The identification of the initial site of the damage will be helpful to identify how HSP27 induces damage.

### 3.5. No Glia Cell Response

Since a degeneration in retina and optic nerve was observed, we also analyzed glia cells. HSP27 acts intracellularly as a chaperone and is generally considered to be protective [57]. In contrast, extracellular HSP27 can activate the immune system via TLRs [42]. Hence, microglia/macrophages might be activated by HSP27 as well. In addition, under stress conditions the level of HSP27 expression was elevated in a microglia culture [58]. The activation of microglia and their harmful effects through the uncontrolled release of proinflammatory cytokines was observed in different glaucoma models [59,60]. However, no differences in the microglia/macrophages number were noted in the current study. Despite that, over 60% of microglia/macrophages could be detected in the GCL, which is understandable as microglia migrate to the site of degeneration [61]. Nevertheless, no altered response of the microglia could be detected in any retinal layer. Also the specific analysis of the microglia population by Tmem 119 antibody showed no significant differences between the HSP27 and the control group. Microglia cells and microglia/macrophages do not seem to react at this stage of degeneration. The evaluated point in time might be too late for a microglia response, which is usually an early event. For example, the intravitreal injection of S100B led to a time dependent activation of the microglia after 14 days [62]. In an ocular hypertension model, with a late onset of the damage, an increased response of microglia was observed after six and 15 weeks [59]. In addition, in DBA/2J mice more microglia were noted after three months of age. The immunization with HSP27 in combination with glial cell line-derived neurotrophic factor (GDNF) also increased the number of microglia, but did not activate them after 4 weeks [63]. However, microglia response could occur at a different point in time after intravitreal HSP27 injection.

Astrocytes are the most common glial cells in the optic nerve head and can enter the retina. After glaucomatous damage, astrocytes strongly react in the retina and the optic nerve of humans [64]. GFAP, the main macroglia protein, was investigated in different glaucoma animal models. The results variate from reduced to significantly increased GFAP levels [65,66,67]. In addition, astrocytes express more HSP27 through stress or external stimuli [68]. In our model, no macrogliosis was detected at 21 days. Possibly, a macroglia response could take place later on. Otherwise, the intravitreal HSP27 injection could trigger an intracellular change of the macroglia and not of their morphology. Right now, we cannot exclude that macroglia were affected at an earlier point in time or later.

## 4. Materials and Methods

### 4.1. Animals

Animal experiments and animal care procedures adhered to the ARVO Statement for Use of Animals in Research and the animal care committee of North Rhine-Westphalia (Germany, 84-02.04.2013.A442; 11 Feb. 2014). In this study, 24 male Wistar rats (376–400 g; Charles River; Sulzfeld, Germany) were involved. All animals had unlimited access to food and water and were kept on a light-dark cycle (12 h:12 h). Detailed health checks and eye exams were performed regularly.

### 4.2. Intravitreal HSP27 Injection

The intravitreal injections of HSP27 or PBS were performed as previously described [34]. Briefly, rats were anesthetized with ketamine (50 mg/mL, Ratiopharm, Ulm, Germany) and xylazine (2%, Bayer Health Care, Leverkusen, Germany). After the pupil was dilated with a mydriatic (Tropicamid 5 mg/mL, Stulln, Germany), a topical anesthetic (Conjuncain 4 mg/mL, Bausch&Lomb, Berlin, Germany) was applied on the eye. The commercially available HSP27 protein (cat. HSP0503; Lot: 097102, AtGen, Yatap-dong, South Korea) was already dissolved in 20 mM Hepesl buffer (pH 7.5). However, to obtain a protein solution of 0.2 µg/µL, we diluted the HSP27 protein in PBS. One eye per animal (*n* = 12) was injected with 2 µL of 0.2 µg/µL (final concentration: 0.4 µg) HSP27 solution under a stereomicroscope (Zeiss, Oberkochen, Germany), with a 32-gauge needle (Hamilton, Reno, NV, USA). Control animals received the same volume of PBS (Biochrome, Berlin, Germany; *n* = 12), since this was used as a solvent for HSP27. After the injection, the eyes were treated with an antibiotic ointment (Floxal, Bausch&Lomb, Berlin, Germany). No injection was performed on the contralateral eye.

### 4.3. Intraocular Pressure Measurement

The IOP of both groups (*n* = 6/group) was measured using a rebound tonometer (TonoLab; Icare, Vantaa, Finland) one day before the intraocular injection (baseline). IOP was also recorded 7, 14, and 21 days after the injection (Figure 1A). Means were calculated from 10 single measurements per eye and point in time.

### 4.4. Electroretinogram Analysis

The ERG measurements (*n* = 6/group) were performed as previously described [34]. Before performing the ERGs under dim red light, rats were dark adapted overnight. In a first step, rats were anesthetized with a mixture of xylazine (2%) and ketamine (50 mg/mL). Additionally, the eyes were treated with mydriatic and conjuncain, and their body temperature was maintained constant at 37 °C by a temperature controller (TC-1000, CWE Inc., Ardmore, PA, USA). To analyze the function of the retina, a full-field flash electroretinograph (HMsERG system; OcuScience, Henderson, NV, USA) was used. Reference electrodes were located under both ears and the ground electrode was positioned subcutaneously in the back over the tail. One drop of methocel (Omni Vision, Puchheim, Germany), a solution for contact glass examinations of the eye, was applied onto the cornea to place recording electrodes combined with a contact lens in the center of both eyes. Before starting the measurement, the function of all electrodes was checked and the faraday cage over the equipment was closed (OcuScience, Henderson, NV, USA). Scotopic flash series with flash intensities at 0.1, 0.3, 1.0, 3.0, 10.0, and 25.0 cd/m^2^ were recorded. Signals obtained from the corneal surface were thereafter amplified, digitized, averaged and stored using ERG View 4.380R software (OcuScience, Henderson, NV, USA). In this case, a low pass filter (150 Hz) was necessary. Amplitudes of a-waves (response of photoreceptor cells) and b-waves (response of cells in the inner nuclear layer) were exported to Excel (Microsoft Corp., Redmond, WA, USA) and then Statistica (V13, Statsoft, Tulsa, OK, USA) for further evaluation.

### 4.5. Preparation of Retina and Optic Nerve

After 21 days, eyes (*n* = 6/group) and optic nerves (*n* = 6/group) were explanted and prepared for histological cross-sections or longitudinal sections, respectively. Eyes were fixed for one hour in 4% paraformaldehyde (Merck, Burlington, MA, USA), whereas optic nerves were fixed for two hours. Afterwards, the tissue was cryo-conserved in 30% sucrose overnight and frozen embedded in NEG-50 Tissue Tek medium (Thermo Fisher Scientific, Cheshire, UK). Retinal cross-sections (10 µm) and longitudinal optic nerve sections (4 µm) were mounted on glass slides (SuperfrostPlus, Thermo Fisher Scientific). Thereafter, the cuts were fixed in ice-cold acetone for 10 min.

For the western blot analysis, 12 eyes (6/group) were obtained. The retina was dissected from the eye bulb and then stored at −80 °C before being processed.

### 4.6. Histological Staining and Following Morphometric Analysis

Structural analyses of retinas (six retinal cross-sections, *n* = 6/group) were done via HE staining (both Merck). Retinal pictures were taken at 200× magnification (four pictures/retina; two peripheral and two central) and retinal thickness was measured from the ganglion cell layer to the outer nuclear layer by using the tool “line” from Zen 2012 software (Zeiss, Oberkochen, Germany). The thickness of the retina was measured three times per retinal image. A total of 12 values were used to calculate the mean value (four pictures/retina; three measurements per picture).

The myelin alterations in the optic nerve were examined using LFB staining (*n* = 6/group). Three pictures per LFB stained optic nerve section (proximal, middle, and distal) were taken at 400× magnification. The LFB stained optic nerves were scored from 0 = intact myelin up to 2 = strong demyelination, in 0.5 intervals [69]. All results were exported to Excel and then Statistica.

### 4.7. Immunofluorescence of Specific Cell Types in Retina and Optic Nerve

Immunofluorescence stainings were performed as previously described [65]. Briefly, six retinal cross-sections (*n* = 6 eyes/group) and six longitudinal optic nerve sections (*n* = 6 nerves/group) were blocked with a mixture of a 10–20% serum, 0.1–0.2% Triton X-100 (Sigma Aldrich, St. Louis, MO, USA), and PBS (Santa Cruz, Dallas, TX, USA). Primary antibodies (Table 1) were diluted in the same blocking mixture and incubated at room temperature overnight. Sections were then stained with Cy3/Alexa Fluor 555 or Alexa Fluor 488 labeled secondary antibodies (Table 1). Cell nuclei were visualized with 4’,6-Diamidin-2-phenylindol (DAPI; Serva Electrophoresis, Heidelberg, Germany). Finally, the sections were covered with Shandon-Mount (Thermo Fisher Scientific). Negative controls were performed by applying only the secondary antibody. Four images per retina (two peripheral and two central) and three per optic nerve (proximal, middle, and distal) were taken using a fluorescence microscope (Axio Imager M1; Carl Zeiss Microscopy) [65]. All pictures were cut to a size of 600/800 pixel using Corel PaintShop Pro (version X8, Corel Corporation, Austin, TX, USA). Evaluation was carried out under masked conditions using ImageJ software (version 1.44p; NIH, Bethesda, MD, USA).

Brn-3a^+^-, PKCα^+^-, calretinin^+^-, or Iba1^+^-cell bodies were counted manually in all pictures. Concerning ED1, the co-localization with Iba1 was counted. In order to detect Iba1^+^ cells with phagocytic activity ED1 antibody was used, and their co-localization with Iba1 was evaluated in the GCL, IPL, and INL separately and in the area from GCL to the INL. To distinguish microglia cells from macrophages, Tmem 119^+^ signals were counted along with Iba1^+^ signals. The co-localized signals of Tmem 119 and Iba1 were counted and evaluated from the GCL to the INL.

GFAP signals in retina and optic nerve were investigated via area analysis using an established ImageJ macro [65]. For this analysis, all retina images were converted into gray scale. After background subtraction (95.8), the lower threshold was set at 7.7 and upper threshold at 70.07 for all retinal sections. In regard to optic nerve sections, background subtraction was 35.8, while the lower threshold was 18.1 and the upper one 77.9 for the GFAP staining.

SMI-32 labeled neurofilaments were scored using an established scoring system ranging from 0 = intact up to 2 = destroyed, in 0.5 intervals [65].

### 4.8. Western Blot Analysis

For western blot analysis, retinas (*n* = 5–6/group) were prepared as previously described [63]. Briefly, for protein isolation, frozen retinas were mechanically homogenized with homogenizer pestle (neoLab, Heidelberg, Germany) in 150 µL lysis buffer (RIPA buffer; Cell Signaling Technology, Danvers, MA, USA) containing protease inhibitor (Sigma-Aldrich). Ultrasound was used for accurate homogenization. Homogenized samples were incubated on ice for 50 min and subsequently centrifuged (30 min; 13,500 rpm; 4 °C). Protein concentrations were determined using a commercial bicinchoninic acid assay (BCA protein assay kit; Thermo Fisher Scientific). Equal amounts of protein (10 or 20 µg) were loaded onto 12% Bis-Tris SDS gels (NuPAGE; Invitrogen, Darmstadt, Germany), and gel electrophoresis was performed at 200 V for 50 min. Proteins were then transferred to a nitrocellulose membrane using the NuPAGE transfer system (Invitrogen). After blocking in 5% w/v milk powder (AppliChem, Darmstadt, Germany) in PBS/0.05% Tween-20 (Sigma-Aldrich), immunostaining was conducted overnight with different primary antibodies and followed the next day by compatible secondary antibodies (Table 1). They were diluted in blocking solution. Protein levels were quantified by densitometry with the Odyssey infrared imager system 2.1 (LI-COR Bioscience, Bad Homburg, Germany). The β-actin protein band was used to normalize the signal intensities. For each analysis, duplets were prepared, and the mean value was used for statistical analyses.

### 4.9. Statistical Analysis

The data were analyzed using Statistica (version 13, Dell Technologies, Round Rock, TX, USA) using a Student’s *t*-test and is presented as mean ± standard error of the mean (SEM). A *p* < 0.05 was considered statistically significant (* *p* < 0.05, ** *p* < 0.01 and *** *p* < 0.001). All results of the PBS control group were set to 100% and compared to the HSP27 group.

## 5. Conclusions

In summary, we noted an IOP-independent loss of RGCs and amacrine cells accompanied by a neurofilament degeneration after intravitreal HSP27 injection. At 21 days, glia cells were not affected. However, it could be too late for a microglia/macrophage response and too early for a macroglia response. Hence, high extracellular HSP27 level caused a neuronal degeneration. As glaucoma patients also have an increased level of HSP27 in the retina, this is of particular interest. Our results might be helpful to understand the role of HSP27 during glaucoma. However, the exact pathomechanisms, leading to the observed neurodegeneration, should be investigated further.

## Figures and Tables

**Table 1:** Primary and secondary antibodies used for immunohistochemistry.

Primary Antibodies			Secondary Antibodies		
Host and Antigen	Company	Dilution	Name	Company	Dilution
*Retinal cross-sections*					
Goat anti-Brn-3a	Santa Cruz	1:100	Donkey anti-goat-Alexa Fluor 488	Abcam	1:400
Goat anti-calretinin	Millipore	1:2000	Donkey anti-goat-Alexa Fluor 488	Dianova	1:500
Rabbit anti-Iba1	Wako	1:500	Goat anti-rabbit-Alexa Fluor 555	Invitrogen	1:400
Mouse anti-ED1	Millipore	1:250	Goat anti-mouse-Alexa Fluor 488	Invitrogen	1:600
Rabbit anti-S100B	Novus biologicals	1:100	Goat anti-rabbit-Alexa Fluor 488	Millipore	1:500
Chicken anti-GFAP	Millipore	1:400	Donkey anti-chicken-Cy3	Millipore	1:500
Mouse anti-PKCα	Santa Cruz	1:300	Donkey anti-mouse-Alexa Fluor 555	Abcam	1:500
Mouse anti-PKCα Mouse anti-Tmem 119	Synaptic Systems	1:200	Donkey anti-mouse-Alexa Fluor 488	Invitrogen	1:600
*Longitudinal optic nerve sections*					
Mouse anti-SMI-32	Convance	1:2000	Goat anti-mouse-Alexa Fluor 488	Invitrogen	1:400
Chicken anti-GFAP	Millipore	1:400	Donkey anti-chicken-Cy3	Millipore	1:500
Rabbit anti-Iba1	Wako	1:400	Goat anti-rabbit-Alexa Fluor 488	Invitrogen	1:500
Mouse anti-ED1	Millipore	1:200	Goat anti-mouse-Alexa Fluor 555v	Invitrogen	1:500
*Western blot*					
Rabbit anti-β-actin	Cell Signaling	1:1000	Donkey anti-rabbit-Dye Light800	Thermo Fisher Scientific	1:20,000
Mouse anti-β-actin	Sigma-Aldrich	1:6000	Donkey anti-mouse-Dye Light 800	LI-COR	1:20,000
Chicken anti-GFAP	Millipore	1:3000	Donkey anti-chicken-IR 680RD	LI-COR	1:20,000
Rabbit anti-Iba1	Synaptic Systems	1:1000	Donkey anti-rabbit-Alexa Fluor 680	Invitrogen	1:5000
Rabbit anti-RBPMS	Millipore	1:200	Donkey anti-rabbit-Alexa Fluor 680	Invitrogen	1:5000

## References

[1] Finger R.P., Fimmers R., Holz F.G., Scholl H.P. (2011). Incidence of blindness and severe visual impairment in germany: Projections for 2030. Investig. Ophthalmol. Vis. Sci..

[2] Casson R.J., Chidlow G., Wood J.P., Crowston J.G., Goldberg I. (2012). Definition of glaucoma: Clinical and experimental concepts. Clin. Exp. Ophthalmol..

[3] Morgan W.H., House P. (2003). Relationship between intraocular pressure and glaucomatous optic neuropathy. Clin. Exp. Ophthalmol..

[4] Tezel G., Yang X., Luo C., Kain A.D., Powell D.W., Kuehn M.H., Kaplan H.J. (2010). Oxidative stress and the regulation of complement activation in human glaucoma. Investig. Ophthalmol. Vis. Sci..

[5] Dreyer E.B., Zurakowski D., Schumer R.A., Podos S.M., Lipton S.A. (1996). Elevated glutamate levels in the vitreous body of humans and monkeys with glaucoma. Arch. Ophthalmol..

[6] Kremmer S., Kreuzfelder E., Klein R., Bontke N., Henneberg-Quester K.B., Steuhl K.P., Grosse-Wilde H. (2001). Antiphosphatidylserine antibodies are elevated in normal tension glaucoma. Clin. Exp. Immunol..

[7] Ikeda Y., Maruyama I., Nakazawa M., Ohguro H. (2002). Clinical significance of serum antibody against neuron-specific enolase in glaucoma patients. Jpn. J. Ophthalmol..

[8] Grus F.H., Boehm N., Beck S., Schlich M., Lossbrandt U., Pfeiffer N. (2010). Autoanti-body profiles in tear fluid as a diagnostic tool in glaucoma. Investig. Ophthalmol. Vis. Sci..

[9] Tezel G., Seigel G.M., Wax M.B. (1998). Autoantibodies to small heat shock proteins in glaucoma. Investig. Ophthalmol. Vis. Sci..

[10] Joachim S.C., Bruns K., Lackner K.J., Pfeiffer N., Grus F.H. (2007). Antibodies to alpha b-crystallin, vimentin, and heat shock protein 70 in aqueous humor of patients with normal tension glaucoma and igg antibody patterns against retinal antigen in aqueous humor. Curr. Eye Res..

[11] Lorenz K., Beck S., Keilani M.M., Wasielica-Poslednik J., Pfeiffer N., Grus F.H. (2017). Course of serum autoantibodies in patients after acute angle-closure glaucoma attack. Clin. Exp. Ophthalmol..

[12] Lindquist S., Craig E.A. (1988). The heat-shock proteins. Annu. Rev. Genet..

[13] Kampinga H.H., Hageman J., Vos M.J., Kubota H., Tanguay R.M., Bruford E.A., Cheetham M.E., Chen B., Hightower L.E. (2009). Guidelines for the nomenclature of the human heat shock proteins. Cell Stress Chaperones.

[14] Bechtold D.A., Brown I.R. (2003). Induction of hsp27 and hsp32 stress proteins and vimentin in glial cells of the rat hippocampus following hyperthermia. Neurochem. Res..

[15] Krueger-Naug A.M., Emsley J.G., Myers T.L., Currie R.W., Clarke D.B. (2002). Injury to retinal ganglion cells induces expression of the small heat shock protein hsp27 in the rat visual system. Neuroscience.

[16] Chidlow G., Wood J.P., Casson R.J. (2014). Expression of inducible heat shock proteins hsp27 and hsp70 in the visual pathway of rats subjected to various models of retinal ganglion cell injury. PLoS ONE.

[17] Tezel G., Hernandez R., Wax M.B. (2000). Immunostaining of heat shock proteins in the retina and optic nerve head of normal and glaucomatous eyes. Arch. Ophthalmol..

[18] Huang W., Fileta J.B., Filippopoulos T., Ray A., Dobberfuhl A., Grosskreutz C.L. (2007). Hsp27 phosphorylation in experimental glaucoma. Investig. Ophthalmol. Vis. Sci..

[19] Sakai M., Sakai H., Nakamura Y., Fukuchi T., Sawaguchi S. (2003). Immunolocalization of heat shock proteins in the retina of normal monkey eyes and monkey eyes with laser-induced glaucoma. Jpn. J. Ophthalmol..

[20] Bruey J.M., Ducasse C., Bonniaud P., Ravagnan L., Susin S.A., Diaz-Latoud C., Gurbuxani S., Arrigo A.P., Kroemer G., Solary E. (2000). Hsp27 negatively regulates cell death by interacting with cytochrome c. Nat. Cell Biol..

[21] Huot J., Houle F., Spitz D.R., Landry J. (1996). Hsp27 phosphorylation-mediated resistance against actin fragmentation and cell death induced by oxidative stress. Cancer Res..

[22] Yokoyama A., Oshitari T., Negishi H., Dezawa M., Mizota A., Adachi-Usami E. (2001). Protection of retinal ganglion cells from ischemia-reperfusion injury by electrically applied hsp27. Investig. Ophthalmol. Vis. Sci..

[23] Chien C.C., Huang C.J., Tien L.T., Cheng Y.C., Ke C.Y., Lee Y.J. (2017). Suppression of hsp27 restores retinal function and protects photoreceptors from apoptosis in a light-induced retinal degeneration animal model. Investig. Ophthalmol. Vis. Sci..

[24] Wax M.B., Tezel G., Yang J., Peng G., Patil R.V., Agarwal N., Sappington R.M., Calkins D.J. (2008). Induced autoimmunity to heat shock proteins elicits glaucomatous loss of retinal ganglion cell neurons via activated t-cell-derived fas-ligand. J. Neurosci. Off. J. Soc. Neurosci..

[25] Joachim S.C., Grus F.H., Kraft D., White-Farrar K., Barnes G., Barbeck M., Ghanaati S., Cao S., Li B., Wax M.B. (2009). Complex antibody profile changes in an experimental autoimmune glaucoma animal model. Investig. Ophthalmol. Vis. Sci..

[26] Batulan Z., Pulakazhi Venu V.K., Li Y., Koumbadinga G., Alvarez-Olmedo D.G., Shi C., O’Brien E.R. (2016). Extracellular release and signaling by heat shock protein 27: Role in modifying vascular inflammation. Front. Immunol..

[27] Rodriguez A.R., de Sevilla Muller L.P., Brecha N.C. (2014). The RNA binding protein RBPMS is a selective marker of ganglion cells in the mammalian retina. J. Comp. Neurol..

[28] Imai Y., Ibata I., Ito D., Ohsawa K., Kohsaka S. (1996). A novel gene iba1 in the major histocompatibility complex class III region encoding an ef hand protein expressed in a monocytic lineage. Biochem. Biophys. Res. Commun..

[29] Bennett M.L., Bennett F.C., Liddelow S.A., Ajami B., Zamanian J.L., Fernhoff N.B., Mulinyawe S.B., Bohlen C.J., Adil A., Tucker A. (2016). New tools for studying microglia in the mouse and human CNS. Proc. Natl. Acad. Sci. USA.

[30] Kuehn S., Grotegut P., Smit A., Stute G., Dick H.B., Joachim S.C. (2018). Imortant role of microglia in a novel s100b based retina degeneration model. Assoc. Res. Vis. Ophthalmol..

[31] Lamprakis I., Todorova M.G., Grub M., Schlote T. (2018). The impact of multiple intravitreal anti-vegf injections on intraocular pressure. Klin. Mon. Augenheilkd..

[32] Kitaoka Y., Kitaoka Y., Kwong J.M., Ross-Cisneros F.N., Wang J., Tsai R.K., Sadun A.A., Lam T.T. (2006). Tnf-alpha-induced optic nerve degeneration and nuclear factor-kappab p65. Investig. Ophthalmol. Vis. Sci..

[33] Manabe S., Lipton S.A. (2003). Divergent NMDA signals leading to proapoptotic and antiapoptotic pathways in the rat retina. Investig. Ophthalmol. Vis. Sci..

[34] Kuehn S., Meißner W., Grotegut P., Theiss C., Dick H.B., Joachim S.C. (2018). Intravitreal s100b injection leads to progressive glaucoma like damage in retina and optic nerve. Front. Cell. Neurosci..

[35] Wagstaff M.J., Collaco-Moraes Y., Smith J., de Belleroche J.S., Coffin R.S., Latchman D.S. (1999). Protection of neuronal cells from apoptosis by hsp27 delivered with a herpes simplex virus-based vector. J. Biol. Chem..

[36] Nahomi R.B., Palmer A., Green K.M., Fort P.E., Nagaraj R.H. (2014). Pro-inflammatory cytokines downregulate hsp27 and cause apoptosis of human retinal capillary endothelial cells. Biochim. Biophys. Acta.

[37] Winningham-Major F., Staecker J.L., Barger S.W., Coats S., Van Eldik L.J. (1989). Neurite extension and neuronal survival activities of recombinant s100 beta proteins that differ in the content and position of cysteine residues. J. Cell Biol..

[38] Kogel D., Peters M., Konig H.G., Hashemi S.M., Bui N.T., Arolt V., Rothermundt M., Prehn J.H. (2004). S100b potently activates p65/c-rel transcriptional complexes in hippocampal neurons: Clinical implications for the role of s100b in excitotoxic brain injury. Neuroscience.

[39] Bianchi R., Kastrisianaki E., Giambanco I., Donato R. (2011). S100b protein stimulates microglia migration via rage-dependent up-regulation of chemokine expression and release. J. Biol. Chem..

[40] Park K.J., Gaynor R.B., Kwak Y.T. (2003). Heat shock protein 27 association with the i kappa b kinase complex regulates tumor necrosis factor alpha-induced NF-kappa b activation. J. Biol. Chem..

[41] Binder R.J., Vatner R., Srivastava P. (2004). The heat-shock protein receptors: Some answers and more questions. Tissue Antigens.

[42] Jin C., Cleveland J.C., Ao L., Li J., Zeng Q., Fullerton D.A., Meng X. (2014). Human myocardium releases heat shock protein 27 (hsp27) after global ischemia: The proinflammatory effect of extracellular hsp27 through toll-like receptor (tlr)-2 and tlr4. Mol. Med..

[43] Gilmore T.D. (2006). Introduction to nf-kappab: Players, pathways, perspectives. Oncogene.

[44] Chen H., Cho K.S., Vu T.H.K., Shen C.H., Kaur M., Chen G., Mathew R., McHam M.L., Fazelat A., Lashkari K. (2018). Commensal microflora-induced t cell responses mediate progressive neurodegeneration in glaucoma. Nat. Commun..

[45] Guiloff G.D., Jones J., Kolb H. (1988). Organization of the inner plexiform layer of the turtle retina: An electron microscopic study. J. Comp. Neurol..

[46] Reinehr S., Kuehn S., Casola C., Koch D., Stute G., Grotegut P., Dick H.B., Joachim S.C. (2018). Hsp27 immunization reinforces AII amacrine cell and synapse damage induced by s100 in an autoimmune glaucoma model. Cell Tissue Res..

[47] Moon J.I., Kim I.B., Gwon J.S., Park M.H., Kang T.H., Lim E.J., Choi K.R., Chun M.H. (2005). Changes in retinal neuronal populations in the dba/2j mouse. Cell Tissue Res..

[48] Hernandez M., Rodriguez F.D., Sharma S.C., Vecino E. (2009). Immunohistochemical changes in rat retinas at various time periods of elevated intraocular pressure. Mol. Vis..

[49] Akopian A., Kumar S., Ramakrishnan H., Viswanathan S., Bloomfield S.A. (2019). Amacrine cells coupled to ganglion cells via gap junctions are highly vulnerable in glaucomatous mouse retinas. J. Comp. Neurol..

[50] Kuehn S., Rodust C., Stute G., Grotegut P., Meissner W., Reinehr S., Dick H.B., Joachim S.C. (2017). Concentration-dependent inner retina layer damage and optic nerve degeneration in a NMDA model. J. Mol. Neurosci..

[51] Cuenca N., Pinilla I., Fernandez-Sanchez L., Salinas-Navarro M., Alarcon-Martinez L., Aviles-Trigueros M., de la Villa P., de Imperial Miralles J., Villegas-Perez M.P., Vidal-Sanz M. (2010). Changes in the inner and outer retinal layers after acute increase of the intraocular pressure in adult albino swiss mice. Exp. Eye Res..

[52] Hartsock M.J., Cho H., Wu L., Chen W.J., Gong J., Duh E.J. (2016). A mouse model of retinal ischemia-reperfusion injury through elevation of intraocular pressure. J. Vis. Exp..

[53] Saggu S.K., Chotaliya H.P., Blumbergs P.C., Casson R.J. (2010). Wallerian-like axonal degeneration in the optic nerve after excitotoxic retinal insult: An ultrastructural study. BMC Neurosci..

[54] Massoll C., Mando W., Chintala S.K. (2013). Excitotoxicity upregulates sarm1 protein expression and promotes wallerian-like degeneration of retinal ganglion cells and their axons. Investig. Ophthalmol. Vis. Sci..

[55] Morrison J.C., Cepurna Ying Guo W.O., Johnson E.C. (2011). Pathophysiology of human glaucomatous optic nerve damage: Insights from rodent models of glaucoma. Exp. Eye Res..

[56] Kim Y.W., Jeoung J.W., Kim D.W., Girard M.J., Mari J.M., Park K.H., Kim D.M. (2016). Clinical assessment of lamina cribrosa curvature in eyes with primary open-angle glaucoma. PLoS ONE.

[57] Franklin T.B., Krueger-Naug A.M., Clarke D.B., Arrigo A.P., Currie R.W. (2005). The role of heat shock proteins hsp70 and hsp27 in cellular protection of the central nervous system. Int. J. Hyperth..

[58] Satoh J.I., Kim S.U. (1995). Differential expression of heat shock protein hsp27 in human neurons and glial cells in culture. J. Neurosci. Res..

[59] Bordone M.P., Gonzalez Fleitas M.F., Pasquini L.A., Bosco A., Sande P.H., Rosenstein R.E., Dorfman D. (2017). Involvement of microglia in early axoglial alterations of the optic nerve induced by experimental glaucoma. J. Neurochem..

[60] Bosco A., Steele M.R., Vetter M.L. (2011). Early microglia activation in a mouse model of chronic glaucoma. J. Comp. Neurol..

[61] Langmann T. (2007). Microglia activation in retinal degeneration. J. Leukoc. Biol..

[62] Grotegut P., Kuehn S., Meissner W., Dick H.B., Joachim S.C. (2019). Intravitreal s100b injection triggers a time-dependent microglia response in a pro-inflammatory manner in retina and optic nerve. Mol. Neurobiol..

[63] Casola C., Reinehr S., Kuehn S., Stute G., Spiess B.M., Dick H.B., Joachim S.C. (2016). Specific inner retinal layer cell damage in an autoimmune glaucoma model is induced by GDNF with or without hsp27. Investig. Ophthalmol. Vis. Sci..

[64] Wang L., Cioffi G.A., Cull G., Dong J., Fortune B. (2002). Immunohistologic evidence for retinal glial cell changes in human glaucoma. Investig. Ophthalmol. Vis. Sci..

[65] Noristani R., Kuehn S., Stute G., Reinehr S., Stellbogen M., Dick H.B., Joachim S.C. (2016). Retinal and optic nerve damage is associated with early glial responses in an experimental autoimmune glaucoma model. J. Mol. Neurosci..

[66] Sun D., Qu J., Jakobs T.C. (2013). Reversible reactivity by optic nerve astrocytes. Glia.

[67] Lam T.T., Kwong J.M., Tso M.O. (2003). Early glial responses after acute elevated intraocular pressure in rats. Investig. Ophthalmol. Vis. Sci..

[68] Nafar F., Williams J.B., Mearow K.M. (2016). Astrocytes release hspb1 in response to amyloid-beta exposure in vitro. J. Alzheimer’s Dis..

[69] Horstmann L., Schmid H., Heinen A.P., Kurschus F.C., Dick H.B., Joachim S.C. (2013). Inflammatory demyelination induces glia alterations and ganglion cell loss in the retina of an experimental autoimmune encephalomyelitis model. J. Neuroinflamm..

